# Left ventricular non-compaction cardiomyopathy associated with the PRKAG2 mutation

**DOI:** 10.1186/s12920-022-01361-2

**Published:** 2022-10-11

**Authors:** Jing Zhang, Xiu Han, Qun Lu, Yunfei Feng, Aiqun Ma, Tingzhong Wang

**Affiliations:** 1 Department of Cardiovascular Medicine, The First Affiliated Hospital of Xi’an Jiaotong University, Xi’an, Shaanxi China; 2 Key Laboratory of Molecular Cardiology, Xi’an, Shaanxi China; 3 Key Laboratory of Environment and Genes Related to Diseases, Xi’an Jiaotong University, Ministry of Education, Xi’an, Shaanxi China

**Keywords:** Left ventricular non-compaction cardiomyopathy, Gene mutation, Next generation sequencing

## Abstract

Left ventricular non-compaction cardiomyopathy (LVNC) is one of the most common inherited cardiovascular diseases. The genetic backgrounds of most LVNC patients are not fully understood. We collected clinical data, family histories, and blood samples and performed genetic analysis using next-generation sequencing (NGS) from a Chinese family of 15 subjects. Clinically LVNC affected subjects showed marked cardiac phenotype heterogeneity. We found that these subjects with LVNC carried a missense heterozygous genetic mutation c.905G>A (p.R302Q) in γ2 subunit of AMP-activated protein kinase (PRKAG2) gene through NGS. Individuals without this mutation showed no symptoms or cardiac structural abnormalities related to LVNC. One subject was the victim of sudden cardiac death. To sum up, PRKAG2 mutation c.905G>A (p.R302Q) caused familial LVNC. Our results described a potentially pathogenic mutation associated with LVNC, which may further extend the spectrum of LVNC phenotypes related to PRKAG2 gene mutations.

**Supplementary Information:**

The online version contains supplementary material available at 10.1186/s12920-022-01361-2.

## Background

Left ventricular non-compaction cardiomyopathy (LVNC) is a rare disease caused by abnormalities in the normal myocardial compaction process. Both sporadic and familial LVNC have been described [1, 2]. In familial disease, LVNC is a genetically heterogeneous disorder [1]. Initial reports showed LVNC can occur either in conjunction with other cardiac diseases or as an isolated phenotype. The true nature and genetic etiology of LVNC, and whether it can be considered a separate disease entity, remains uncertain. Although diagnosis focuses primarily on the identification and description of trabeculae, other characteristics are critical to defining specific subtypes of LVNC. They have a wide range of features [3, 4]. It was reported that there are at least eight different phenotypes of LVNC seem to exist [5], including benign LVNC, LVNC with arrhythmias, dilated LVNC, hypertrophic LVNC, hypertrophic dilated LVNC, restrictive LVNC, right ventricular or biventricular LVNC, LVNC with congenital heart disease. Of these, the hypertrophic subtype, hypertrophic LVNC, is characterized by left ventricular hypertrophy, often accompanied by asymmetrical interventricular septal hypertrophy, diastolic dysfunction and hypercontractile systolic function [6]. The prognosis of patients with this subtype appears to be similar to that of the general population or those with hypertrophic cardiomyopathy (HCM) without LVNC.

Mutations in the γ2 subunit of AMP-activated protein kinase (PRKAG2) gene are known to cause an energetic disease affecting the myocardium [7] and previous studies have confirmed that they can cause Wolff-Parkinson-White (WPW) syndrome, HCM, ventricular pre-excitation and very few symptoms of myopathy [7–9]. The PRKAG2 gene encodes the γ_2_ subunit of the AMP-activated protein kinase (AMPK) [10] which may be involved in cardiac development, particularly in the development of the atrioventricular (AV) annulus fibrosus [11–14]. AMPK is a highly conserved kinase responsible for the control of the cellular energetic balance [15]. In the cardiomyocyte, AMPK is implicated in promoting myocardial fatty acid uptake [16], oxidation [17], glucose uptake [18], glycolysis [19, 20], and possibly glycogen storage or exercise [21, 22], all of which may contribute to the maintenance of an adequate supply of ATP necessary for normal cardiac function. The main feature of myocardial histopathology in PRKAG2 cardiomyopathy is extensive intracellular vacuoles filled with glycogen [7, 23, 24].

In the present study, we studied a Chinese family in which the proband presented with the hypertrophic LVNC and confirmed for the first time that PRKAG2 mutation c.905G>A (p.R302Q), previously proposed to be associated with HCM and WPW syndrome [7–9], was associated with LVNC.

## Methods

### Clinical evaluation

Available detailed clinical evaluation of the proband and her relatives was performed, including an accurate medical history, physical examination, 12-lead electrocardiogram (ECG), echocardiography and cardiac magnetic resonance (CMR). Due to the distance from the hospital or their young age, several subjects did not visit the hospital for clinical examinations. CMR procedures were performed by using a 3.0T scanner (GE Healthcare). A 17-segment model was made from three short axis sections. According to American Heart Association criteria [25], the presence or absence of non-compaction and was qualitatively assessed using the 17 segment model. The ratio of non-compacted to compacted (NC/C) myocardium was measured on three long-axis views for each involved myocardial segment in diastole, and the maximum ratio was then used for analysis. Noncompaction was defined as a ratio of NC/C on end-diastole > 2.3 [26]. Sinus bradycardia was defined as a heart rate less than 60 beats per minute (bpm), and short PR interval was defined as a PR interval < 120 ms on electrocardiogram.

## Samples

Blood samples were collected from the preexisting witnesses and their families. Genomic deoxyribonucleic acid (DNA) was extracted from peripheral blood using a QIAmp DNA blood mini kit (Qiagen, Limburg, NL) according to the standard procedures. Qubit was used for accurate quantification of DNA concentration.

## Next generation sequencing and analysis

Genomic DNA were randomly broken into 150-200 bp fragments by Bioruptor Pico ultrasound, and fragmented DNA was end-repaired and “A” added at the 3’ end. splice ligation. Next, sample labeling and enrichment of DNA were performed by PCR amplification. Libraries with specific indexes were further mixed for capturing using TargetSeq® liquid chip capture sequencing kits with Biotin-labeled RNA probe, and then using stranded affinity-labeled magnetic spheres to obtain target gene exons, followed by PCR amplification for target gene enrichment. Library quantification was performed using Qubit 3.0, and concentration > 25ng/ul was considered as qualified library. The main peak of the library should be around 220-320 bp with no spurious peaks before and after the main peak by Agilent 2100 assay. Illumina NextSeq 500 sequencing platform was used for sequencing after the quantification of qualified libraries. The raw image data files obtained from high-throughput sequencing were transformed into raw sequenced sequences (Sequenced reads). The Sequenced reads were then aligned to the reference sequences or reference genomes (GRCh37/hgl9). Clean reads were obtained by preliminary filtering of the Sequenced reads. The Burrows-Wheeler Aligner tool was used to compare the clean reads with the reference genome, and the sequencing work was performed to obtain the bam result file, and the average library length, comparison rate, coverage rate, capture rate, sequencing depth, homogeneity and other indicators were analyzed. All the mutations were compared with databases such as dbSNP, ExAC or 1000 genome projects. All the mutations found that can potentially cause cardiac ion channelopathies and cardiomyopathy were subject to Sanger sequencing (ABI3730xl) for verification.

## Conservation analysis and bioinformatics prediction

The homologs of the region including Arg302 in homo sapiens were detected (HomoloGene, http://www.ncbi.nlm.nih.gov/homologene). The potential pathogenicity of the identified missense mutation was evaluated by combining different methods: PolyPhen-2 (http://genetics.bwh.harvard.edu/pph2), SIFT (http://sift.jcvi.org), and Mutation Taster (http://www.mutationtaster.org). The reference protein ID of PRKAG2 was ‘Q9UGJ0 ’ and the Ensembl transcript ID was ‘ENSG00000106617’.

## Results

### Clinical features of the proband

**Fig. 1:**
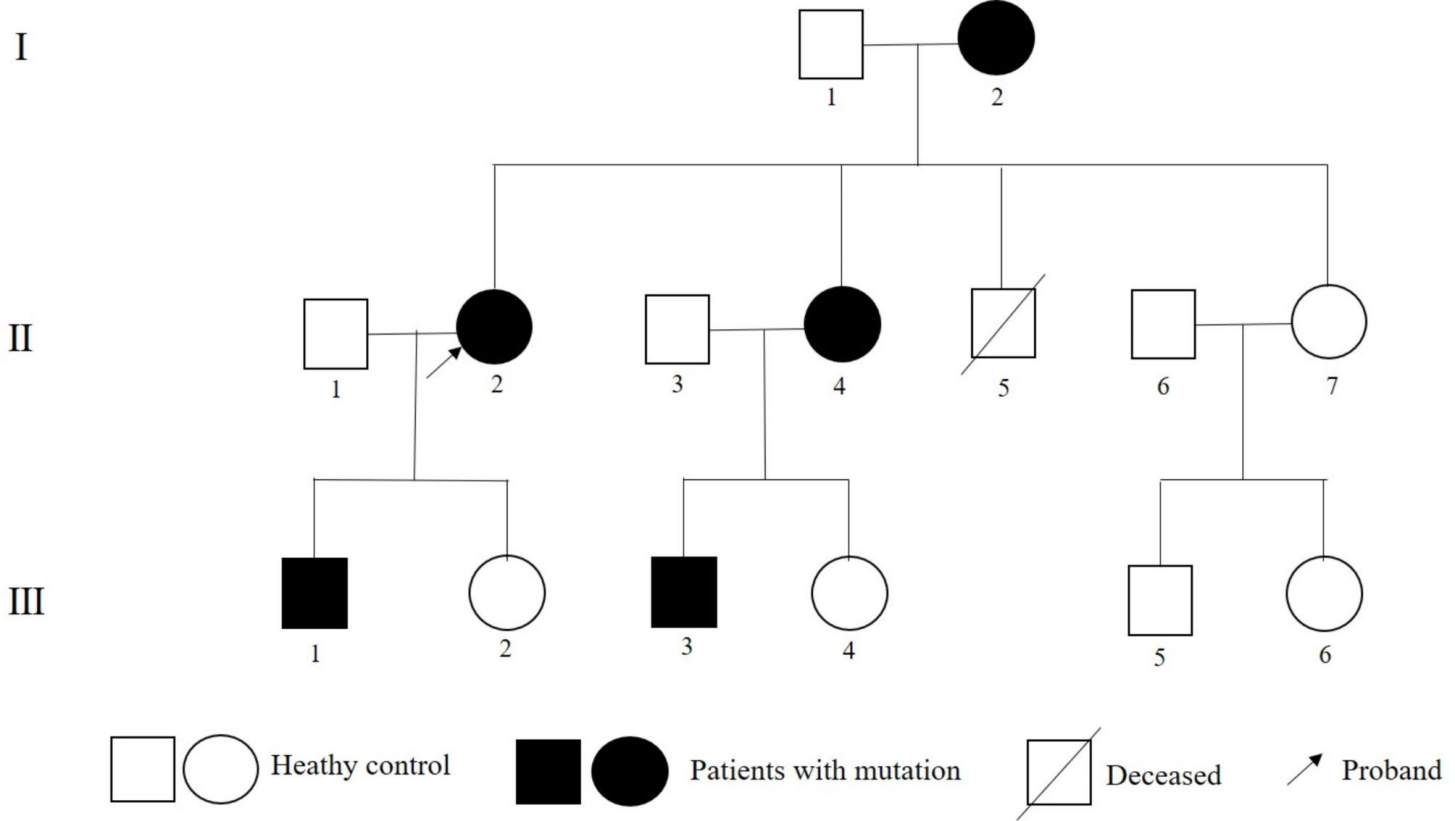
Pedigree structure of the family. Family members are identified by generations and numbers. Squares, male family members; circles, female members

The proband of this family was a 36-year-old female (II-2, Table 1; Fig. 1). She had paroxysmal palpitations and chest distress for about 6 months, without angina pectoris, dyspnea or syncope. Physical examination revealed that grade II /III systolic murmurs could be heard at the left lower sternal border due to mitral regurgitation.

**Table 1:** Clinical Characteristics of the Family Members

Age	Gender	PRKAG2 mutation c.905G>A	ECG	Echocardiography				CMR	Symptoms	Comments
				**IVST (mm)**	**LVEDD (mm)**	**LVPWT (mm)**	**EF (%)**			
64	M	No	Normal	9	50	8	66	-	No	
63	F	Yes	Dual chamber pacing	7	43	8	63	-	Palpitation and chest distress	Dual-chamber pacemaker
36	M	No	Normal	9	45	9	64	-	No	No
36	F	Yes	Sinus bradycardia, short PR interval and LVHV	15	46	11	65	LVNC	Palpitation and chest distress	No
29	M	No	Normal	9	51	9	76	-	No	No
30	F	Yes	Sinus bradycardia, short PR interval	7	45	7	69	LVNC	Palpitation	No
24^*^	M	No	-		-	-	-	-	-	Sudden cardiac death
39	M	-	-		-	-	-	-	No	No
38	F	-	Normal		-	-	-	-	No	No
12	M	Yes	Normal	6	42	5	64	LVNC	No	No
6	F	No	Normal	5	33	4	64	-	No	No
6	M	Yes	Normal	6	35	5	71	-	No	No
2	F	-	-					-	No	No
19	M	-	-		-	-	-	-	No	No
3	M	-	-		-	-	-	-	No	No

^*^ Subject II-5 died at the age of 24. ECG, electrocardiogram; LVHV, left ventricular high voltage; IVST, interventricular septum thickness; LVEDD, left ventricular end diastolic diameter; LVPWT, left ventricular posterior wall thickness; EF, ejection fraction; CMR, cardiac magnetic resonance; LVNC, left ventricular non-compaction cardiomyopathy

**Fig. 2:**
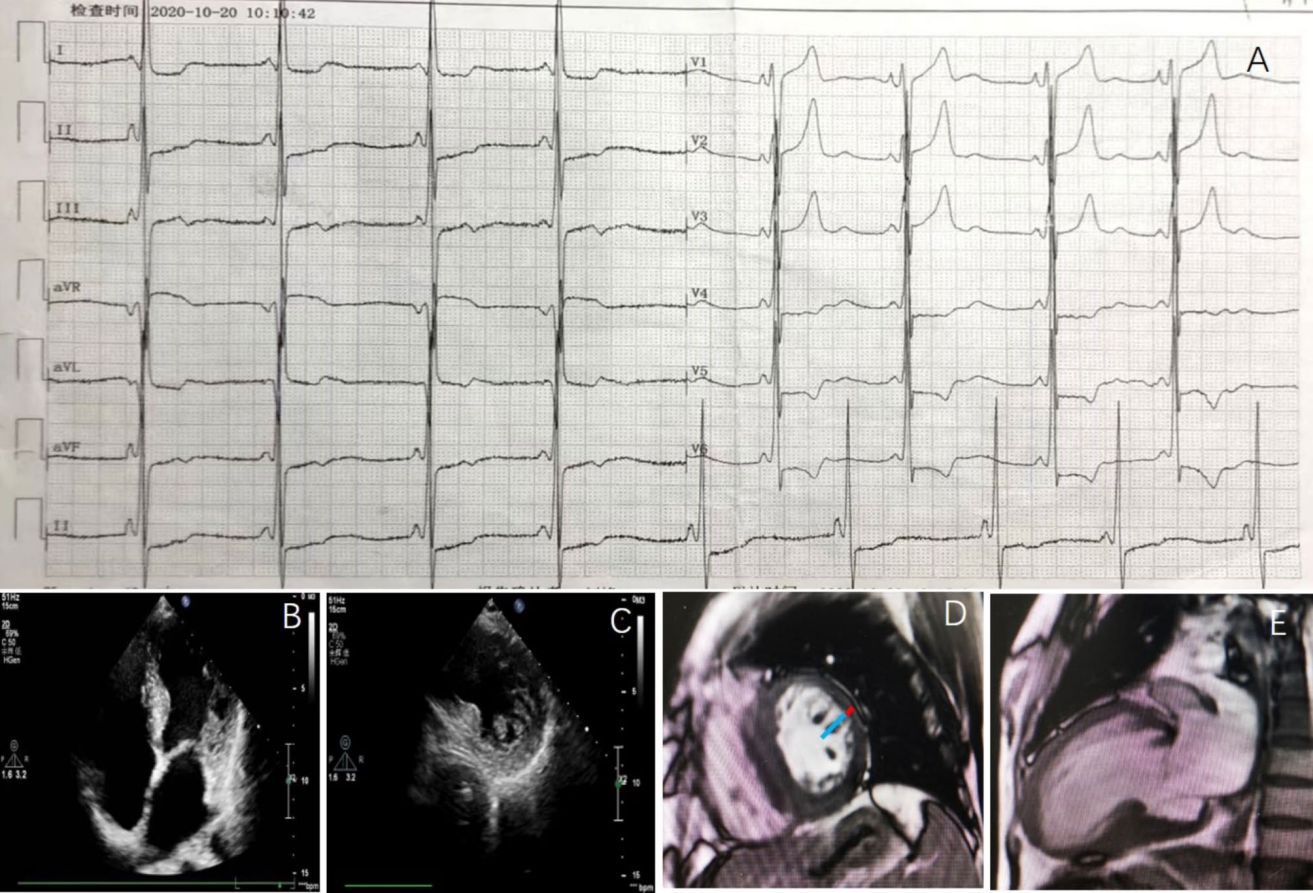
The electrocardiogram, transthoracic echocardiography, and cardiac magnetic resonance of the proband. **A:** The 12-lead electrocardiogram showed sinus bradycardia, short PR interval and left ventricular high voltage. **B:** Echocardiographic apical 4-chamber view showed uneven hypertrophy of the ventricular septum with the basal segment being the thickest. There are abundant myocardial trabeculae in the lateral walls of the mid-segment and apical portions of the left ventricle. **C:** Echocardiographic short axis view indicated prominent myocardial trabeculae in the apical portion of the left ventricle, forming a loose network. **D:** Cardiac magnetic resonance image of short axis at the level of the apical segments showed abundant trabeculation overlying a very thin compacted myocardial layer in the lateral left ventricular wall. Blue bar: non-compacted wall thickness; red bar: compacted wall thickness. **E:** Cardiac magnetic resonance image of long axis 2-chamber projection showed the layer of non-compact myocardium.

The proband’s ECG showed sinus bradycardia (around 53 bpm), short PR interval (114 ms) and left ventricular high voltage with nonspecific ST-T wave changes (Fig. 2 A). Her echocardiography showed that the left atrium was enlarged (43 × 64 mm) with a small amount of mitral regurgitation. The left ventricle (LV) wall was unevenly thickened, mainly in the septum, with the basal segment of the septum thickened by about 15 mm and the middle segment by about 13 mm, the posterior wall of the left ventricle by about 11 mm and the lateral wall of the left ventricle by about 12 mm, with normal systolic amplitude (Fig. 2B). The lateral wall of the LV middle segment and the lateral wall of the apical part of the heart were rich and thickened in myocardial trabeculae, forming a loose network (Fig. 2 C). The CMR image indicated that the ratio of NC/C on end-diastole in the lateral wall of the LV middle segment and the apical part were greater than 2.3. There were abundant trabeculation overlying a very thin compacted myocardial layer in the lateral left ventricular wall (Fig. 2D and E).

## Pedigree analysis

The proband’s brother (II-5) died of sudden cardiac death (SCD) suddenly at age 24. Unfortunately, the records of her brother were not available to us. Her mother (I-2) was hospitalized 10 years ago for sinus bradycardia, complete right bundle branch block, left anterior branch block, Lown-Ganong-Levine syndrome and underwent radiofrequency ablation of the atrioventricular node bypass. Unfortunately, she developed third degree AV block after the procedure, so she underwent a dual-chamber pacemaker implantation. Her repeat ECG revealed intermittent atrial flutter and complete right bundle branch block 6 years ago. Her young sister (II-4) had varying degrees of palpitations and chest distress. Her ECG showed sinus bradycardia, short PR interval. Her echocardiogram and CMR showed LVNC. The proband’s son (III-1) had no clinically significant symptoms. His ECG showed normal, but the echocardiogram and CMR showed LVNC. Subject III-3 was too young to cooperate with CMR test. Subjects II-6, II-7, III-4, III-5, III-6 did not present at our hospital due to the long distance to the hospital, so their blood samples and clinical documents could not be obtained (Table 1; Fig. 1).

## Mutation identification

**Fig. 3:**
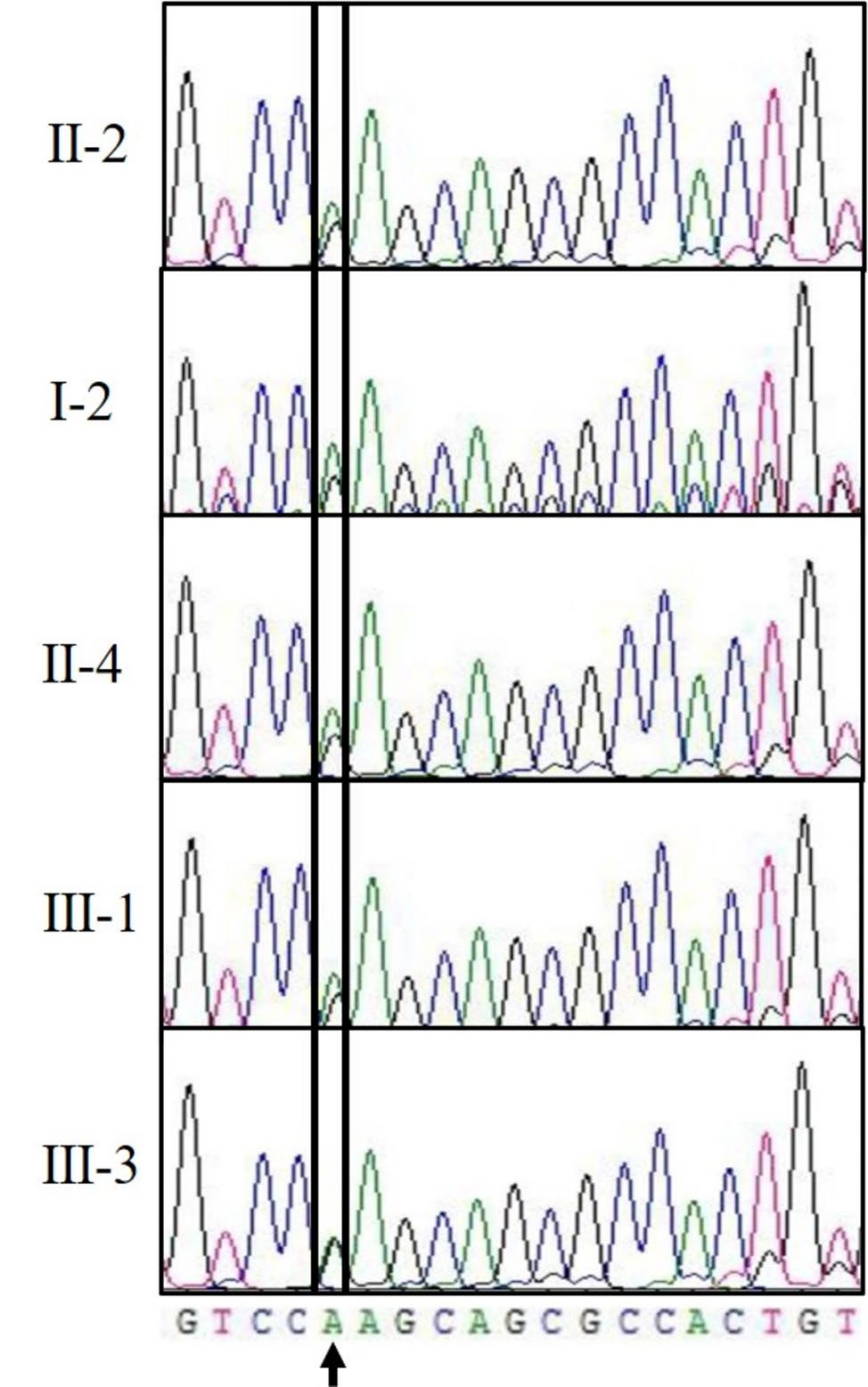
A disease-causing mutation of PRKAG2 in LVNC family, black arrow indicated missense variant c.905G>A.

To identify the genetic basis of the LVNC suffered by this family, a custom-made next-generation sequencing (NGS) panel consisting of 675 genes previously associated with cardiomyopathies and related striated muscle disorders was used (Supplemental Table 1). Genetic sequencing was performed in the proband (II-2) using this NGS panel, and the mutation c.905G>A was found in PRKAG2 (RefSeq: NM_016203), corresponding to a nonsynonymous amino acid change from arginine to glutamine at position 302 (p.R302Q) (Fig. 3). Next, the above mutation was sequenced in the other family members. The PRKAG2 mutation c.905G>A (p.R302Q) was confirmed in the subjects (I-2, II-4, III-1 and III-3) and was not seen in other asymptomatic family members. The proband’s nephew (III-3) who had no clinical symptoms currently was also found to carry the mutation.

## Discussion

In the present study, we studied the family in which the proband presented with the hypertrophic LVNC. We performed NGS on the family members and identified a missense heterozygous genetic mutation in PRKAG2 [c.905G>A (p.R302Q)] which might to be the potentially pathogenic mutation. To our knowledge, this is the first study to describe the association of this mutation with LVNC.

LVNC, first described by Grant in 1926 [27], is a heterogeneous myocardial disease characterized by prominent trabeculae, intratrabecular recesses, and two distinct layers of left ventricular myocardium: compaction and non-compaction [28, 29]. There is continuity between the left ventricular cavity and the deep intratrabecular recesses [2]. Assessment of imaging and pathologic changes shows that the disease is characterized by spongy left ventricular myocardium with abnormal trabeculae, usually most pronounced at the left ventricular apex [30]. The development of LVNC is associated with cessation of end-stage myocardial compaction and morphogenesis [2, 31–33].

The American Heart Association (AHA) classifies LVNC as a separate genetic cardiomyopathy, but the European Society of Cardiology (ESC) defines it as an unclassified entity [34]. It is still controversial whether LVNC is a unique cardiomyopathy or a common morphological feature of different types of cardiomyopathies. It was demonstrated that LVNC may exist in isolation or in conjunction with other cardiomyopathies. There have been reported that LVNC may combined with glycogen storage disease [35], Fabry disease [36], Danon disease [37] and so on. Sometimes, LVNC is not initially accompanied by other cardiomyopathies. Some cardiomyopathies exist in isolation in the early stages. However, late-onset LVNC usually subsequently occurs after experiencing several non-genetic factors including increased cardiac preload or other abnormal load conditions that lead to disruption of cardiac homeostasis [38].

Among familial diseases, LVNC is a genetically heterogeneous disease that usually shares mutations in genes encoding for sarcomere proteins [1]. Currently, the incidence of LVNC is increasing, which may be due to greater awareness and more sensitive diagnostic tools such as modern ultrasound techniques and CMR [3, 39–41]. CMR has improved cardiac imaging, which has allowed for a more detailed understanding of the disease [30]. More importantly, genetics and genetic analysis play an important role in the prediction and management of LVNC [42]. However, due to the lack of a true gold standard, LVNC is likely to be overdiagnosed, which may challenge the differentiation of LVNC from other cardiomyopathies. Therefore, it is necessary to further describe the morphological characteristics of LVNC.

The proband’s echocardiography showed uneven thickening of the left ventricular wall, most notably in the basal segment of the ventricular septum. The lateral wall of the left ventricle middle segment and the lateral wall of the apical part of the heart were rich in myocardial trabeculae and were increased and thickened. Previous trials have collected a total of 242 children diagnosed with isolated LVNC, and 66 patients (27%) presented with hypertrophic [43]. Hypertrophic LVNC is often difficult to distinguish from HCM. We can distinguish them from the following three aspects. First, the thickness ratio between trabecular myocardium and normal myocardium does not reach more than 2 in hypertrophic LVNC. Second, the trabecular region associated with hypertrophic LVNC tends to be segmental rather than diffuse like left ventricular hypertrophy [44]. Thirdly, the myocardial wall trabecular in LVNC is most often located at the apex of the heart and the lateral wall and lower wall of LV [45]. These differences in segmental prevalence suggest that the normal process of myocardial densification from the basal septum to the apical lateral segment is interrupted early in development.

The proband and her young sister presented with short PR and sinus bradycardia. PRKAG2 cardiac syndrome may include conduction system disease, and an increased risk of SCD. It was reported that the short PR interval was largely attributed to excessive glycogen accumulation in myocytes and presented in 68% of PRKAG2 syndrome patients [46]. Sinus bradycardia was also associated with LVNC [28, 47]. Previous studies have confirmed that patients misdiagnosed with HCM were definitively LVNC by autopsy, and eight of nine patients had arrhythmias, in most cases sinus bradycardia, confirming that ventricular muscle dyssynchrony was often associated with conduction defects [45, 48, 49]. It has been proposed that fibrosis might be a possible cause of AV in patients with LVNC [50, 51]and defective local myocardial angiogenesis may be a potential cause of conduction abnormalities. Patients with progressive sinus bradycardia are associated with an abnormal vascular supply near the sinus node. Our study indicated that except for one SCD victim (II-5), the most LVNC survivors were about more than 30 years old. The mechanisms of how this missense mutation leads to LVNC are not clear yet. Further biochemical and cell biological studies are needed to determine the consequence of this missense mutation.

The proband’s sister and son (II-4 and III-1) manifesting with LVNC carried the mutation. The proband’s brother (II-5) died of SCD, thus we concluded that the proband’s brother may have suffered from LVNC and carried the same mutation. Our predictive genetic testing identified an asymptomatic individual (III-3) who was 6 years old and carried the disease-causing mutation, but unfortunately, he was so young that he could not cooperate to complete the CMR. His associated clinical symptoms have not yet expressed. Therefore, on the basis of our genetic diagnosis of LVNC in this family, we will facilitate better disease management and follow-up by clinicians before the appearance of symptoms. Bioinformatic prediction can provide us with some useful information about the pathogenicity of the PRKAG2 mutation c.905G>A (p.R302Q). However, our experiments do not reflect the true pathology of this mutant in cardiac myocytes. Due to the inaccessibility of human heart tissues, we were unable to obtain sufficient numbers of patient heart tissues. The creation of mutant mice using CRISPR/Cas9 gene editing methods or the use of patient-specific induced pluripotent stem cell (iPSC) derived cardiomyocytes are state-of-the-art methods to directly and reproducibly study the pathogenicity of human mutants. In the future, we will further investigate the pathogenic role of the mutation using cardiomyocytes derived from patient-specific iPSCs.

## Conclusion

We reported a missense heterozygous mutation of PRKAG2 [c.905G>A (p.R302Q)] in a Chinese family that presented with LVNC. The present study confirmed the genotype-phenotype correlation of the PRKAG2 mutation and provided further insight into the genetic factors underlying LVNC pathology. In addition, our findings demonstrate that CMR and NGS assays are effective methods for identifying pathogenic mutations associated with hereditary cardiomyopathies.

## Electronic supplementary material

Below is the link to the electronic supplementary material.

Supplementary Material 1

## Data Availability

The data are not publicly available as the considerations about the security of human familial genetic resources and the confidentiality of participants, but they can be obtained from the corresponding author on reasonable request.
