# Supplementary material for: Left ventricular non-compaction cardiomyopathy associated with the PRKAG2 mutation
Source: BMC Med Genomics. 2022 Oct 11;15:214. doi: 10.1186/s12920-022-01361-2 (PMC9552423; doi:10.1186/s12920-022-01361-2)
Supplement: Supplementary file 1 — Supplementary Material 1 [file 12920_2022_1361_MOESM1_ESM.pdf]

Left ventricular non-compaction cardiomyopathy associated with the PRKAG2 mutation

Jing Zhang [1, 2, 3], Xiu Han [1], Qun Lu [1], Yunfei Feng[1, 2, 3], Aiqun Ma [1, 2, 3*], Tingzhong Wang [1, 2, 3*]

[1] Department of Cardiovascular Medicine, The First Affiliated Hospital of Xi'an Jiaotong University, Xi'an, Shaanxi, China

[2] Key Laboratory of Molecular Cardiology, Xi'an, Shaanxi, China

[3] Key Laboratory of Environment and Genes Related to Diseases, Xi'an Jiaotong University, Ministry of Education, China

*Corresponding authors at: Department of Cardiovascular Medicine, The First Affiliated Hospital of Xi'an Jiaotong University, No. 277 Yanta West Road, Xi'an 710061, Shaanxi, China.

E-mail addresses: aiqun.ma@xjtu.edu.cn (A. Ma), tingzhong.wang@xjtu.edu.cn (T. Wang)

Supplemental Table. Detailed Information of NGS Gene Panel for Cardiovascular Diseases (675 Genes)

Cardiovascular diseases	Gene name
Ion channelopathies and cardiomyopathies	A2ML1、AARS2、ABCA1、ABCC2、ABCC6、ABCC9、ABCD3、ABCD4、ACAD9、ACADL、ACADM、ACADS、ACADVL、ACAT1、ACOX1、ACSF3、ACTA1、ACTC1、ACTN2、AGA、AGK、AGL、AGL1、AGPS、AGXT、AIFM1、AKAP9、ALDOA、ALG1、ALG11、ALG12、ALG13、ALG2、ALG3、ALG6、ALG8、ALG9、ALPK3、ALSM1、AMACR、AMPD1、ANK2、ANKRD1、ANOS1、APOPT1、ARFGEF2、ARSA、ARSB、ARSE、ARX、ASAH1、ATP5A1、ATP5E、ATP6V0A2、ATP6VOA2、ATP7A、ATP7B、ATPAF2、ATRX、B3GAT3、B4GALT1、BAG3、BCS1L、BMP1、BMP2、BRAF、BTD、C10ORF2、C12orf65、CACNA1C、CACNA1S、CACNA2D1、CACNB2、CACNB2I2、CALM1、CALM2、CALM3、CALR3、CASQ2、CAV3、CBL、CBS、CBX2、CCDC115、CD320、CDH2、CDKL5、CDKN1C、CEP89、CFL2、CHRM2、CHST14、CHST3、CHST6、CHSY1、CIT、CLPB、CLPP、CNBP、COA3、COA5、COA6、COA7、COG1、COG2、COG4、COG5、COG6、COG7、COG8、COL1A1、COL1A2、COSMC、COX10、COX14、COX15、COX20、COX4、COX4I2、COX6A、COX6A1、COX6B、COX6B1、COX7B、COX8、COX8A、CPT1A、CPT2、CREB3L1、CRTAP、CRYAB、CSRP3、CTF1、CTNNA3、CTSA、CVL、CYC1、CYP11A1、D2HGDH、DDOST、DES、DGUOK、DHCR24、DHCR7、DHDDS、DHH、DLAT、DLD、DMD、DMPK、DMRT1、DNAJC19、DNASE1L1、DNM2、DOLK、DPAGT1、DPM1、DPM2、DPM3、DSC2、DSG2、DSP、DTNA、DUFA4、EARS2、EBP、ECHS1、ECI1、ECSIT、EFTA、ELAC2、EMD、ENO3、EPM2A、EPM2B、ETFA、ETFB、ETFDH、ETHE1、EXT1、EXT2、EYA1、EYA4、FAH、FARS2、FASTKD2、FBN1、FBN2、FBXL4、FGF12、FHL1、FHL2、FKBP10、FKRP、FKTN、FLAD1、FLNC、FOXD4、FOXRED1、FRDAFXN、FUCA1、FUCT1、FUT8、FXN、G6PC、G6PC3、GAA、GAD1、GALC、GALE、GALK、GALNS、GALNT12、GALNT3、GALT、GATA4、GATA6、GATAD1、GBA、GBE1、GCK、GCS1、GFER、GFM1、GFM2、GFPT1、GGCX、GJA5、GLA、GLB1、GLUT2、GM2A、GMPPA、GNB2、GNE、GNPAT、GNPTAB、GNPTAG、GNPTG、GNS、GPAM、GPD1L、GRHPR、GSTZ1、GTPBP3、GUSB、GYG1、GYG2、GYS1、GYS2、H19、HADH、HADHA、HADHB、HAMP、HARS2、HBB、HCCS、HCFC1、HCN4、HEXA、HEXB、HEY2、HFE、HFE2、HGOA1、HGSNAT、HIBCH、HMGCL、HOGA1、HOPX、HPD、HRAS、HSD3B2、HYAL1、IARS2、ICR1、IDH2、IDS、IDUA、IFITM5、IGF2、ILK、ITGA7、IVD、JPH2、JUP、KBTBD13、KCNAB2、KCNB2、KCND2、KCND3、KCNE1、KCNE2、KCNE3、KCNE5、KCNH2、KCNH2TV3、KCNJ16、KCNJ18、KCNJ2、KCNJ5、KCNQ1、KCNQ1OT1、KIF21B、KLF10、KLHL40、KLHL41、KRAS、LAMA2、LAMA4、LAMP2、lamp2b、LARS2、LBR、LDB3、LDHA、LFNG、LIAS、LINC00850、LIPA、LIPT1、LMBRD1、LMNA、LMOD3、LRPPRC、LRRC10、LYRM7、LZTR1、MAGT1、MAMLD1、MAN1B1、MAN2B1、MANBA、MAP2K1、MAP2K2、MAP3K1、MAP3K8、MAT1A、MCCC2、MCEE、MCOLN1、MGAT2、MGST3、MIB1、MLYCD、MMAA、MMAB、MMACHC、MMADHC、MOG1、MOGS、MPDU1、MPI、MPS7、MPV17、MRPS34、MRPS7、MRRF、MTFMT、MTHFR、MTM1、MTR、MTRR、MTTP、MURC/CAVIN4、MUT、MVK、MYBPC3、

	MYH6、MYH7、MYH7B、MYL2、MYL3、MYLK2、MYOM1、MYOZ2、MYPN、MYST4、NAGA、NAGLU、NARS2、NDFUA11、NDUF1V、NDUFA1、NDUFA10、NDUFA11、NDUFA12、NDUFA13、NDUFA2、NDUFA3、NDUFA4、NDUFA5、NDUFA6、NDUFA7、NDUFA8、NDUFA9、NDUFAB1、NDUFAF1、NDUFAF2、NDUFAF3、NDUFAF4、NDUFAF5、NDUFAF6、NDUFAF7、NDUFB1、NDUFB10、NDUFB11、NDUFB2、NDUFB3、NDUFB4、NDUFB5、NDUFB6、NDUFB7、NDUFB8、NDUFB9、NDUFC1、NDUFC2、NDUFS1、NDUFS1TV2、NDUFS2、NDUFS3、NDUFS4、NDUFS5、NDUFS6、NDUFS7、NDUFS8、NDUFV1、NDUFV2、NDUFV3、NEB、NEBI3、NEBL、NEU1、NEXN、NFKB1、NIPSNAP1、NIPSNAP3A、NKX2-5、NKX2- 5、NNT、NOS1AP、NPC1、NPC2、NPL、NPPA、NR0B1、NR5A1、NRAP/AR、NRAS、NSDHL、NUBPL、NUS1、OBSCN、OPA1、OXCT1、P3H1、PCCA、PCCB、PDHA1、PDHB、PDHX、PDLIM3、PDSS2、PET100、PET117、PEX1、PEX11B、PEX12、PEX2、PEX26、PEX3、PEX5、PEX6、PEX7、PFKM、PGAM2、PGM1、PHKA1、PHKA2、PHKB、phkbtv2、PHKG2、PHYH、PI4KA、PIGA、PIGM、PIGN、PIGV、PKP2、PLEC、PLEKHM2、PLN、PLOD2、PMM2、PMP22、PNPT1、POLG、POMGNT1、POMT1、POMT2、PPIB、PRDM16、PRDM8、PRDX1、PRKAG2、PSAP、PSEN1、PSEN2、PTCD1、PTPN11、PTRH2、PYGL、PYGM、QRSL1、RAF1、RANGRF、RASA2、RBM20、RFT1、RIMS1、RIT1、RNF207/CLORF188、RRAS、RRM2B、RYR1、RYR2、RYR3、SBDS、SC5D、SCN10A、SCN1B、SCN2B、SCN3B、SCN4A、SCN4B、SCN5A、SCNN1A、SCO1、SCO2、SDHA、SDHAF1、SDHB、SDHD、SEC24D、SEMA3A、SERAC1、SERPINF1、SERPINH1、SGCA、SGCB、SGCD、SGCG、SGSH、SHOC、SHOC2、SIRT6、SIX1、SIX5、SLC17A3、SLC19A3、SLC22A5、SLC25A10、SLC25A19、SLC25A20、SLC25A4、SLC25A46、SLC2A5、SLC35A1、SLC35A2、SLC35C1、SLC35D1、SLC35G2、SLC37A4、SLC39A8、SLC40A1、SLC4A3、SLC52A1、SLC52A2、SLMAP、SMN1、SMN2、SMPD1、SNTA1、SOD2、SOS1、SOS2、SOX9、SPARC、SPEG、SPRY1、SRD5A3、SRY、SSR4、ST3GAL3、STAR、STT3A、STT3B、SUCLA2、SUCLG1、SUCLG2、SUGCT、SUN2、SURF1、SYNE1、SYNE2、SYNM、TACO1、TAT、TAZ、TBX20、TBX3、TBX5、TCAP、TECRL、TENT5A、TF、TFAM、TFAP2A、TFR2、TGFB3、TIMM50、TIMMDC1、TK2、TKT、TMEM126B、TMEM15、TMEM165、TMEM199、TMEM38B、TMEM43、TMEM70、TMPO、TNNC1、TNNI3、TNNI3K、TNNT1、TNNT2、TNNT3、TPK1、TPM1、TPM2、TPM3、TRDN、TRIM63、TRMU、TRPM4、TSFM、TTC19、TTN、TTR、TUFM、TUSC3、TWNK、TXNRD2、TYMP、UBR4、UQCC2、UQCC3、UQCRB、UQCRC2、UQCRQ、VCL、VIL1、VPS33A、WNT1、WNT4、WT1、WWOX、XGPT1、XIRP1、xirp2tv1、YWHAE、ZBTB17、ZNF9
